# Panoramic Observation of Crystalline Lenses with 25 MHz Ultrasonography

**DOI:** 10.1155/2019/8319027

**Published:** 2019-11-11

**Authors:** Wenwen Xue, Haidong Zou

**Affiliations:** ^1^Department of Ophthalmology, Shanghai Eye Disease Prevention and Treatment Center, Shanghai Eye Hospital, Shanghai 200040, China; ^2^Department of Ophthalmology, Shanghai General Hospital, Shanghai Jiao Tong University School of Medicine, Shanghai 200080, China

## Abstract

**Purpose:**

To visualize and assess in vivo the age-related changes in crystalline lens size and contour.

**Methods:**

Seventy-nine healthy volunteers, 39 females and 40 males, with a mean age of 41.53 + 11.32 years (range: 21 to 60 years) were enrolled in this study. The axial lens thickness (ALT), equatorial lens diameter (ELD), and anterior (*R*_a_) and posterior (*R*_p_) lens surface radii of curvatures of the subjects' left eyes were measured with a 25 MHz ultrasound probe.

**Results:**

The mean ALT and ELD were 4.178 mm + 0.288 mm and 9.209 mm + 0.214 mm, respectively. There was a statistically significant increase in both ALT (slope = 11 *μ*m/year, *r* = 0.88, *p* < 0.01) and ELD (slope = 6 *μ*m/year, *r* = 0.60, *p* < 0.01) with age. *R*_a_ negatively correlated, and *R*_p_ did not change with age.

**Conclusion:**

There were no statistically significant relationships between any studied values and gender. Independent of gender, the lens grows equatorially and axially with age while its central anterior lens surface steepens and its posterior central surface curvature does not change.

## 1. Introduction

Understanding the normal functioning of the human lens and its role in the development of refraction, accommodation and presbyopia requires a thorough knowledge of how lens size and contour change with age. The central 1 mm-to-6 mm zone of the lens within the pupillary area is easily visualized. Axial lens thickness (ALT), central lens radius of curvature, can be measured using anterior segment optical coherence tomography or Scheimpflug photography. Although these devices have proven to provide high resolution and valid in vivo images of the lens of the eye, none of these devices can visualize the contour of the lens equator because the iris blocks the penetration of light. Although 50 MHz ultrasound biomicroscopy can visualize a small portion of the lens equator, only magnetic resonance imaging (MRI) has been able to visualize the entire contour of the lens including its equator [1–3]. However, MRI has low resolution and is infrequently used in the field of ophthalmic clinical observation because it is time-consuming and expensive.

In this study a 25 MHz B-scan ultrasound device was used to visualize and assess the age-related changes in the entire lens contour including ALT and ELD and the radii of curvatures of the central anterior and posterior lens surfaces.

## 2. Materials and Methods

### 2.1. Subjects

This healthy volunteer prospective study was completed within 1 year (January 1, 2014, to December 1, 2015). The inclusion criteria were the following: Chinese Han race; 21–60 years of age; no history of systematic diseases, such as hypertension, diabetes, or other diseases; best-corrected visual acuity 6/6 or higher in both eyes; and a refractive error between −3.00 D and +3.00 D and otherwise normal ophthalmic examination. Children or teenagers were not enrolled because of their intolerance to corneal-contact examinations. Volunteers with eye diseases, such as cataracts, glaucoma, retinal diseases, or strabismus, and in whom the equator of the lens was not clearly identifiable with the 25 MHz B-scan ultrasound probe were excluded. All volunteers received a routine eye examination without mydriasis that included best-corrected visual acuity, auto-refraction (RM-8900, Topcon, Tokyo, Japan), biomicroscopy, and ophthalmoscopy. This investigation complied with the Declaration of Helsinki and was approved by the Institutional Ethical Board at the Shanghai General Hospital, Shanghai Jiao Tong University. All examination procedures were clearly explained to the subjects, and informed consent was obtained.

### 2.2. Ultrasonography

**Figure 1:**
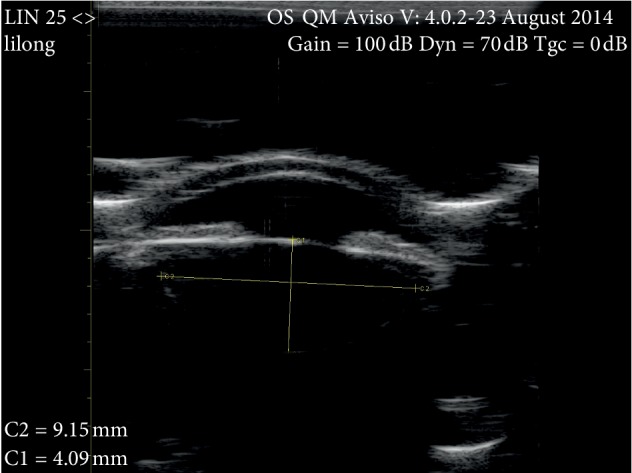
A 25 MHz ultrasonographic image of the left eye of a 40-year-old female. The gain was set to 100 dB, the dynamic range to 70 dB, and the time gain compensation to 0 dB. The entire contour of the lens, including the equator anterior and posterior surfaces, was clearly visible. Using the calipers, yellow lines, shown in the image, the axial lens thickness and equatorial lens diameter were calculated as 4.09 mm (C1) and 9.15 mm (C2), respectively.

**Figure 2:**
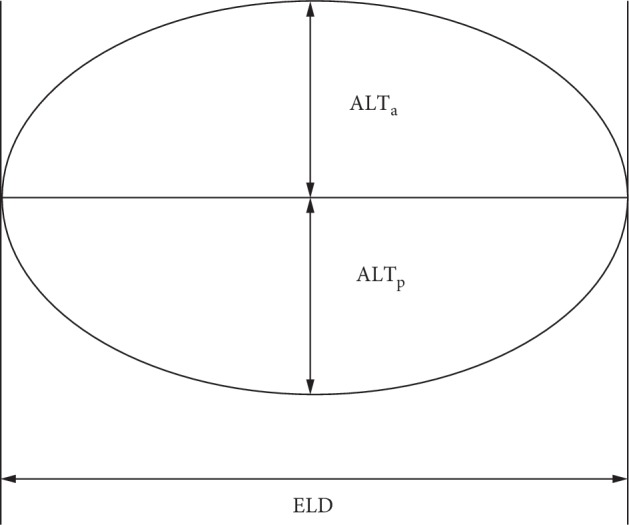
A schematic drawing of a lens. ELD represents the equatorial lens diameter, ALT_a_ represents the anterior part of axial lens thickness, and ALT_p_ represents the posterior part of the axial lens thickness.

A 25 MHz B-scan ultrasonography device (AVISO Diagnostic Ultrasonography, Quantel Medical, France) was used to visualize the entire contour of the lens. In previous studies, this device has been shown to make highly reliable repeatable measurements [4]. The axial and lateral resolutions of the 25 MHz B-scan ultrasonographic probe are estimated to be 60 *μ*m and 120 *μ*m, respectively. Two experienced ophthalmologists (HZ and WX) conducted the following procedures. Each subject was placed in the supine position on an examination table in natural light. After a topical anesthetic drop was administered to the left eye, the 25 MHz B-scan probe ultrasound probe with an attached water bladder was gently placed vertically onto the center of the left cornea. Starting from the 12 o'clock position, the probe was rotated once 360°. The gain, dynamic range, time-gain compensation, contrast, and intensity of the ultrasound were adjusted to ensure maximum visualization of the entire left lens including its equator while the subject stared with the right eye at a 3 m high ceiling. An ultrasound image was frozen when the whole equator of the lens was clearly observed, as shown in Figure 1. The caliber of the ultrasound device was used to measure 3 times the ELD, anterior (ALT_a_), and posterior (ALT_p_) lens thicknesses as shown in Figure 2. In addition, a 10 MHz A-scan ultrasonographic probe (AVISO Diagnostic Ultrasonography, Quantel Medical, France) was used to measure ALT. The 10 MHz probe was gently placed in contact with the center of the left cornea while the subject fixated at the light within the center of the probe.

### 2.3. Analysis

The central 1 to 3 mm of the lens anterior and posterior surfaces were assumed to be spherical, and the radius of the central anterior lens surface was calculated using the following formula:

(1) $$\begin{array}{c} R_{\text{a}}=\left[\frac{{\text{ELD}}^{2}}{8*{\text{ALT}}_{\text{a}}}\right]+0.5*{\text{ALT}}_{\text{a}}. \end{array}$$

The curvature of the central anterior surface (*K*_a_) was calculated as the reciprocal of *R*_a_. The radius (*R*_p_) and curvature (*K*_p_) of the central posterior lens surface were calculated with the same method.

### 2.4. Statistics

Consistency between the ALT results measured by 25 MHz B-scan and 10 MHz A-scan ultrasonography were analyzed using an intragroup coefficient (ICC). Correlations between ALT and ELD were evaluated using Pearson's correlation analysis. The relation between studied values and gender were assessed with the Student's *t*-test. A statistical package (SPSS V10.0, Chicago, IL, USA) was used for database setup and analysis. The level of statistical significance was set at *p* < 0.05.

## 3. Results

### 3.1. Subjects

One hundred and fifty-seven healthy volunteers were screened; however, 78 subjects were excluded because their lens equators were too fuzzy to identify; i.e., only 79 subjects (50.3%) met the inclusion criteria. Of the 79 enrolled subjects, 40 (50.63%) were male and 39 (49.37%) were female. The average age was 41.53 ± 11.32 years old. There were 17 (21.5%) subjects in the 21-to-30-year-old age group, 20 (25.3%) in the 31-to-40-year-old age group, 21 (26.6%) in the 41-to-50-year-old age group, and 21 (26.6%) in the 51-to-60-year-old age group. The refractive error of the left eye of these 79 subjects was between −2.00 D and +2.50 D.

### 3.2. Measurements

The mean (standard deviation) of the 25 MHz B-scan ultrasonographic ALT, ELD, *R*_a_, *K*_a_, *R*_p_, and *K*_p_ was 4.178 mm (+0.288 mm), 9.209 mm (+0.214 mm), 10.499 mm (+0.975 mm), 0.096/mm (+0.009/mm), 4.981 mm (+0.135 mm), and 0.201/mm (+0.005/mm), respectively. There were no statistically significant gender differences in these measurements as shown in Table 1.

The 10 MHz A-scan ultrasonography measured ALT was 4.168 mm (+0.291 mm). An intragroup correlation analysis of the ALT measured by the 25 MHz B-scan and 10 MHz A-scan probes showed that the two techniques were highly statistically significantly consistent (ICC = 0.962, *p* < 0.01).

The ALT, ELD, *R*_a_, *K*_a_, *R*_p_, and *K*_p_ using 25 MHz B-scan ultrasonography of the 79 eyes are shown in Table 2.

### 3.3. Correlations

**Figure 3:**
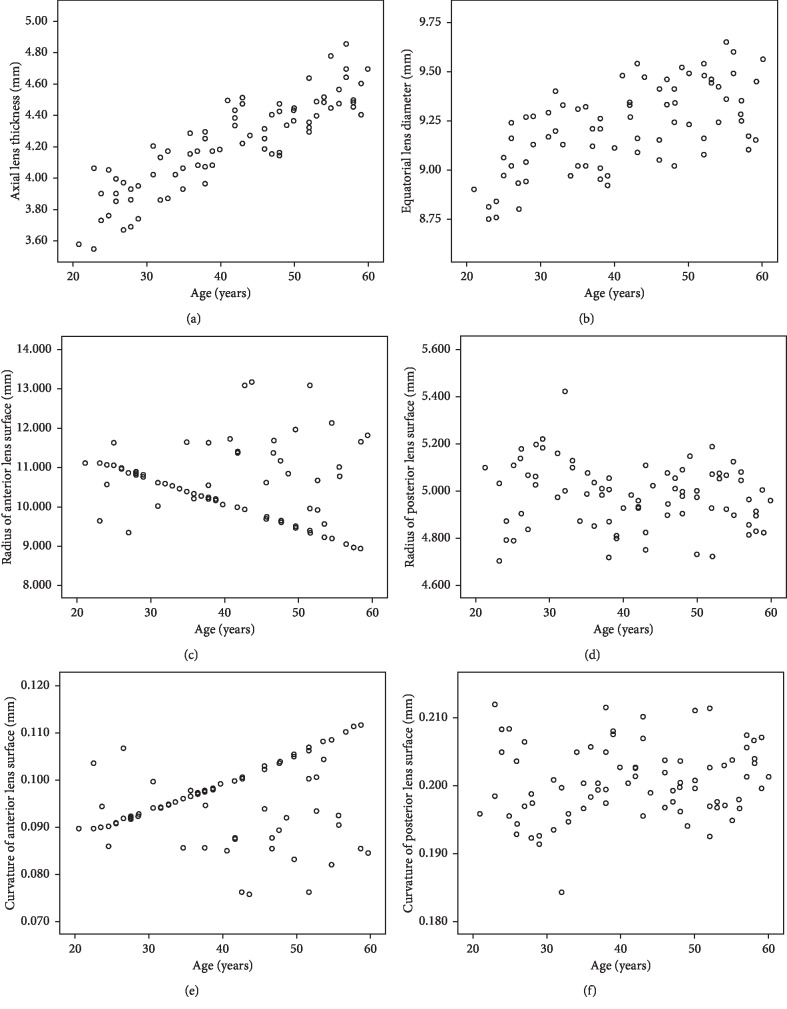
Scatter plots of ALT, ELD, radius, and curvature of the central anterior and posterior lens surface versus age for the entire 79 healthy left eyes using 25 MHz B-scan ultrasonography.

A positive correlation was found between age and ALT and ELD (slope = 11 *μ*m/year and 6 *μ*m/year, Pearson's correlation coefficient, *r* = 0.880, *p* < 0.01 and *r* = 0.600, *p* < 0.01, respectively). There was a negative correlation between age and *R*_a_. There was no statistically significant correlation between age and *R*_p_ or *K*_p_ (*p* > 0.05) as shown in Figure 3.

## 4. Discussion

In the present study, a panoramic observation of the entire crystalline lens contour was obtained with a 25 MHz B-scan ultrasonography. This technique has relatively high axial and lateral resolutions estimated to be 60 *μ*m and 120 *μ*m, respectively. The accuracy of the measurements in the present study was confirmed by the highly statistically significant correlation between the 25 MHz B-scan and 10 MHz A-scan probe ALT measurements. However, even though the technique has good resolution, 50% of subjects screen failed because the lens equator contour was ambiguous. Possible reasons for the difficulty in imaging the equatorial region in these screened failed subjects were poor fixation with their right eyes or that their pupils were naturally more dilated resulting in a thicker peripheral iris causing a decrease in penetration of the ultrasound.

In the 79 enrolled subjects, there was a statistically significant non-gender-related and age-related increase in both ALT and ELD and a decrease in *R*_a_; however, *R*_p_ did not change with age. Other studies have also confirmed an age-related increase in ALT [2, 5, 6, 7]. For example, and similar to the present study, Atchison et al. found with A-scan ultrasonography an age-related ALT increase of 0.0235 mm/year in 106 emmetropes aged 18 to 69 years [2]. And with optical coherence tomography (Lenstar LS900, Haag-Streit Diagnostics, Köniz, Switzerland), Adnan et al. also reported a similar age-related increase in ALT of 0.020 mm/year [6].

The observed age-related increase in ELD is consistent with *in vitro* ELD [8, 9] and *in vivo* MRI measurements [2, 3]. Using Scheimpflug photography, Dubbelman and Heijde found that the radius of curvature of the central 3 mm zone of the anterior lens surface decreases with age according to the following equation: *R*_a_ = 12.9–0.057 *∗* age [10]. Similar to the present study Atchison et al. found that there was not a statistically significant decrease in *R*_p_. Consistent with the present study, previous studies found that the age-related decrease in *R*_a_ was not gender dependent [2, 10].

Some inherent limitations of the present study should be noted. First, the observed subjects were in a single race over a limited age range. Second, since all subjects were asked to stare at the 3 m high ceiling, it was assumed that during all measurements the subjects were not or only minimally accommodating. Consistent with this assumption, the mean *R*_a_ = 10.5 mm (range: 8.97 to 11.82 mm). Third, in the present study, it was assumed that the lens is axisymmetric; however, Atchinson et al. found with MRI that the horizontal and vertical lens equatorial diameters slightly differ [2].

## 5. Conclusions

ALT and ELD increase with age while the *R*_a_ decreases and the *R*_p_ does not change. Gender does not appear to affect the size or contour of the lens. The age-related increase in ELD is consistent with Schachar's theory of presbyopia [11] and probably plays a role in altering the stress on the lens resulting in cortical cataracts [12].

## Figures and Tables

**Table 1:** Dimensions by gender of 79 crystalline lenses measured with a 25 MHz B-scan ultrasonic probe.

	Males	Females	Student's *t*-test	*p* value
Axial lens thickness (mm)	4.190 ± 0.285	4.167 ± 0.295	0.35	0.727
Equatorial lens diameter (mm)	9.216 ± 0.193	9.202 ± 0.236	0.293	0.77
Radius of anterior lens surface (mm)	10.513 ± 0.977	10.485 ± 0.985	0.125	0.901
Radius of posterior lens surface (mm)	4.980 ± 0.128	4.982 ± 0.143	0.083	0.934
Curvature of anterior lens surface (mm)	0.096 ± 0.009	0.096 ± 0.009	0.123	0.902
Curvature of posterior lens surface (mm)	0.201 ± 0.005	0.201 ± 0.006	0.059	0.953

Note. Data are presented as mean ± standard deviation.

**Table 2:** Mean axial lens thickness, equatorial lens diameter, radius, and curvature of the anterior and posterior lens surfaces of each of the 79 eyes measured by 25 MHz B-scan ultrasonography.

Age (years)	Number	Axial lens thickness (mm)	Equatorial lens diameter (mm)	Anterior lens surface		Posterior lens surface	
				Radius (mm)	Curvature (mm)	Radius (mm)	Curvature (mm)
21	1	3.54	8.9	11.112	0.09	5.099	0.196
23	2	3.765	8.78	10.379	0.097	4.87	0.206
24	2	3.775	8.8	10.825	0.093	4.832	0.207
25	2	3.865	9.015	11.344	0.088	4.948	0.203
26	3	3.873	9.14	10.977	0.091	5.073	0.197
27	2	3.78	8.865	10.107	0.1	4.952	0.202
28	3	3.787	9.083	10.849	0.092	5.093	0.197
29	2	3.805	9.2	10.788	0.093	5.202	0.193
31	2	4.07	9.23	10.321	0.097	5.067	0.198
32	2	3.955	9.3	10.596	0.094	5.211	0.192
33	2	3.98	9.23	10.534	0.095	5.115	0.196
34	1	3.98	8.97	10.463	0.096	4.871	0.205
35	2	3.955	9.165	11.022	0.091	5.032	0.199
36	2	4.175	9.17	10.274	0.098	4.944	0.203
37	2	4.085	9.165	10.263	0.098	4.997	0.201
38	4	4.103	9.108	10.659	0.094	4.913	0.204
39	2	4.085	8.945	10.181	0.098	4.805	0.208
40	1	4.14	9.11	10.062	0.099	4.926	0.203
41	1	4.45	9.48	11.734	0.085	4.981	0.201
42	3	4.34	9.313	10.926	0.092	4.939	0.203
43	3	4.36	9.263	10.985	0.093	4.894	0.205
44	1	4.23	9.47	13.179	0.076	5.021	0.199
46	3	4.207	9.203	10.021	0.1	4.974	0.201
47	2	4.235	9.395	11.534	0.087	5.032	0.199
48	4	4.258	9.253	10.011	0.101	4.992	0.201
49	1	4.29	9.52	10.849	0.092	5.146	0.194
50	3	4.37	9.237	10.319	0.098	4.901	0.204
52	4	4.358	9.315	10.446	0.097	4.977	0.201
53	2	4.395	9.45	10.3	0.098	5.063	0.198
54	2	4.455	9.33	9.396	0.107	4.993	0.2
55	2	4.565	9.505	10.668	0.096	5.012	0.2
56	2	4.475	9.545	10.904	0.092	5.062	0.198
57	3	4.687	9.293	9.054	0.11	4.877	0.205
58	3	4.433	9.147	8.971	0.111	4.879	0.205
59	2	4.46	9.3	10.3	0.099	4.912	0.204
60	1	4.65	9.56	11.816	0.085	4.959	0.202

## Data Availability

The data used to support the findings of this study are available from the corresponding author upon request.

## Abbreviations

ALT: Axial lens thickness
ALT_a_: Anterior portion of the axial lens thickness
ALT_p_: Posterior portion of the axial lens thickness
ELD: Equatorial lens diameter
*K*_a_: Anterior lens surface curvature
*K*_p_: Posterior lens surface curvature
*R*_a_: Anterior lens surface radius of curvature
*R*_p_: Posterior lens surface radius of curvature.
